# Implementation of inclusion and exclusion criteria in clinical studies in OHDSI ATLAS software

**DOI:** 10.1038/s41598-023-49560-w

**Published:** 2023-12-18

**Authors:** Romina Blasini, Kornelia Marta Buchowicz, Henning Schneider, Birgit Samans, Keywan Sohrabi

**Affiliations:** 1 Institute of Medical Informatics, Justus Liebig University, Giessen, Germany; 2 Faculty of Health Sciences, University of Applied Sciences, Giessen, Germany

**Keywords:** Translational research, Population screening

## Abstract

Clinical trials are essential parts of a medical study process, but studies are often cancelled due to a lack of participants. Clinical Trial Recruitment Support Systems are systems that help to increase the number of participants by seeking more suitable subjects. The software ATLAS (developed by Observational Health Data Sciences and Informatics) can support the launch of a clinical trial by building cohorts of patients who fulfill certain criteria. The correct use of medical classification systems aiming at clearly defined inclusion and exclusion criteria in the studies is an important pillar of this software. The aim of this investigation was to determine whether ATLAS can be used in a Clinical Trial Recruitment Support System to portray the eligibility criteria of clinical studies. Our analysis considered the number of criteria feasible for integration with ATLAS and identified its strengths and weaknesses. Additionally, we investigated whether nonrepresentable criteria were associated with the utilized terminology systems. We analyzed ATLAS using 223 objective eligibility criteria from 30 randomly selected trials conducted in the last 10 years. In the next step, we selected appropriate ICD, OPS, LOINC, or ATC codes to feed the software. We classified each criterion and study based on its implementation capability in the software, ensuring a clear and logical progression of information. Based on our observations, 51% of the analyzed inclusion criteria were fully implemented in ATLAS. Within our selected example set, 10% of the studies were classified as fully portrayable, and 73% were portrayed to some extent. Additionally, we conducted an evaluation of the software regarding its technical limitations and interaction with medical classification systems. To improve and expand the scope of criteria within a cohort definition in a practical setting, it is recommended to work closely with personnel involved in the study to define the criteria precisely and to carefully select terminology systems. The chosen criteria should be combined according to the specific setting. Additional work is needed to specify the significance and amount of the extracted criteria.

## Introduction

The field of medicine is eternally linked to study goals to discover more about the human body in an effective and positively influential way. The ultimate aim of a medical study is always to improve patient care and therapeutic standards throughout clinical trials when applied. To observe the influence of new interventions and prevention strategies in terms of safety and efficiency, a certain number of participants are recruited according to some specified criteria. Such a recruitment process is always a challenging and time-consuming stage of the medical study pipeline, resulting in a large body of cancelled or incomplete studies^1–3^.

Eligibility criteria (EC) are established at the outset of each clinical trial to describe the shared characteristics of participating individuals. Patient cohorts are typically identified and recruited by clinical or primary care personnel who query clinical systems manually for eligible patients. This process is costly and time-intensive, constituting an important component of the clinical research study pipeline. Therefore, utilizing digital tools can substantially enhance subject recruitment and decrease associated expenses and necessary labor resources^4^.

Clinical Trial Recruitment Support Systems (CTRSSs), also known as patient recruitment systems (PRSs), can bolster patient inclusion in clinical trials by automatically analyzing eligibility criteria based on electronic health records^4–6^. Although these systems are currently increasingly integrated into many medical research projects, most study centers do not tend to use such digital tools for patient recruitment^7^.

The primary challenge is the need for technical staff and equipment to execute implementation and supportive tasks for the CTRSS. One possible solution is to deploy a dedicated system that functions across multiple platforms or to incorporate the CTRSS into existing research systems. In addition to the existing CTRSS or commercial solutions developed as part of different projects, several open-source tools, such as the *Observational Medical Outcomes Partnership Common Data Model* (OMOP CDM) developed by *Observational Health Data Sciences and Informatics* (OHDSI) or *Informatics for Integrating Biology and the Beside* (i2b2) established by the *National Institutes of Health* (NIH), are used in multisite research projects. Both systems are based on the idea of using standard terminologies within data repositories; however, they are not limited to a particular one. In addition, data can be extracted and harmonized from different sources, so they may be utilized in different medical contexts and locations. On the user interface of these systems, it is possible to define criteria, which are used to filter the data and therefore search for certain patient cohorts^8,9^.

In a previous work by Reinecke et al., they described an architecture for a CTRSS that technically integrates OMOP CDM as the data basis and ATLAS as a graphical user interface for formalizing eligibility criteria^10,11^.

### Observational health data sciences and informatics (OHDSI)

The OMOP CDM is part of a whole tool suite created and maintained by OHDSI as a collaboration among 150 organizations worldwide that collect and process health care data. OHDSI offers a wide range of open-source tools to support various data analytics use cases on observational patient-level data, all of which interact with databases using the Common Data Model^8,12^.

Inspired by OHDSI, developers created the open-source web-based software application known as ATLAS. Its primary function is to serve as a user interface for the OMOP CDM and to facilitate observational analysis by extracting patient data from clinical practice for generating real-world evidence. The software enables the definition of cohorts, selection of analysis configurations, and application of appropriate codes to tag diseases. Additionally, it allows for easy project sharing among researchers^8,10^.

ATLAS can also be used for subject recruitment by formalizing the eligibility criteria of a trial into the corresponding cohort^10^. The definition of trials in ATLAS works with two main designs: cohorts and concepts. Concepts are individual codes that belong to a terminology system integrated in the OMOP CDM. They can be grouped into concept sets. A concept set contains several concepts from the standardized vocabulary in combination with logical indicators. The set allows the user to specify related concepts that should be included or excluded in the vocabulary hierarchy. Cohorts are a collection of individuals searched for by a particular query based on specific criteria, so all of the trial eligibility criteria are part of the cohort definition^8^.

### Terminology systems

The application of terminology systems forms an essential fundament when using digital tools because they are unambiguous compared to natural language, which minimizes the risk for misunderstandings or nonmatching subjects. A terminology system offers a comprehensive and standardized definition of terms utilized in a specific field and the associated linguistic expressions, including synonymous terms^13,14^. The OMOP Common Data Model (CDM) requires that data ingestion adhere to vocabularies, which are essentially terminology systems embedded within the OMOP CDM. Standard vocabularies, such as Logical Observation Identifiers Names and Codes (LOINC) or Systematized Nomenclature of Medicine Clinical Terms (SNOMED CT), are supported by default in OMOP CDM. Furthermore, it is feasible to incorporate additional nonstandard vocabularies. Usually, all codes of these nonstandard vocabularies are mapped to one or more codes of the standard vocabularies^8,15,16^.

Several analyses have been conducted to investigate the use of digital systems based on international terminologies^17,18^. For example, Chondrogiannis et al. investigated the use of a *Clinical Data Interchange Standard Consortium* (CDISC)-based model in conjunction with a patient-centered model to structure eligibility criteria for clinical studies. International classification systems, such as the *International Classification of Diseases* (ICD), formed the basis for this project. They found that the majority of the criteria could be represented in the system^19^.

When conducting digital searches for potential clinical trial participants in local hospitals, researchers use the patients' electronic health record (EHR) data that are already available. The success of this search depends on the terminology system utilized for documentation within the hospital staff's workflow.

Some studies have integrated eligibility criteria with OMOP CDM. Automated tools such as criteria2query implement natural language processing (NLP) to formalize eligibility criteria into ATLAS cohort definitions. The latter method is effective when working with standard OMOP vocabularies. Although more studies have been conducted on this issue, there remains a gap between theory and practical implementation of eligibility criteria using specific terminology systems in software^17,20^. Therefore, further analysis should be conducted regarding this matter to explore the hidden potentials behind the different software and coding systems. This study considered the possibility of applying the inclusion and exclusion criteria of different clinical trials in ATLAS as a proof of concept.

## Objectives

The objective of this study was to investigate the representation of eligibility criteria in ATLAS. Furthermore, this study aimed to identify the obstacles in the implementation of the aforementioned criteria. This also included the technical difficulties in the implementation process as well as the qualitative drawbacks. The secondary care data were connected to data available in the EHRs of the patients. Therefore, we decided to define the search parameters with the terminology systems that were already locally in use. We selected terminology systems that are widely used in Germany. First, the German modifications of the standard classification systems ICD, Tenth Revision (ICD-10-GM) and the *International Classification of Procedures in Medicine* (ICPM) named the *Operation and Procedure Code (OPS)* in Version 2021 were used. These systems are utilized for billing in all German hospitals and are therefore widely used. Additionally, the *Anatomical Therapeutic Chemical classification* (ATC) Version 2021 was selected for the medications, and LOINC Version 2.71 was selected as the coding system for laboratory results.

As a secondary objective, we wished to determine to what extent and which impact the selection of terminology systems had on the results. Therefore, we considered whether implementation would be possible with the help of SNOMED CT. We used SNOMED CT version 2021-01-31.

This research focused on classification systems and standards, as well as dealing with the mapping of inclusion and exclusion criteria generally and in ATLAS specifically. Therefore, this work might be interesting for experts involved in subject recruitment phases while applying digital solutions in this area.

## Methods

### Study design

To complement our research, we randomly chose 30 studies across various medical disciplines. To select these studies, we searched on the platform "ClinicalTrials.gov", one of the largest registries of clinical trials including approximately 425,817 studies from 222 different countries^21^. The selected studies had to have been started within the past 10 years, resulting in 114 studies, among which 30 studies were chosen randomly.

To assess the feasibility of implementing eligibility criteria in ATLAS, we compiled a table documenting 331 criteria from 30 studies. We then categorized these criteria based on Luo et al.'s semantic categories, excluding redundancies^22^.

### Procedure in ATLAS

**Figure 1:**
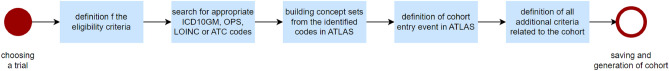
Procedure of portraying a trial in ATLAS software.

To start the criteria introduction procedure in ATLAS, we selected an appropriate terminology system with fitting codes for each eligibility criterion, as shown in Fig. 1. The suitable codes were identified using official online catalogs^15,23–25^.

If no appropriate codes were found in these catalogs, SNOMED CT was used in the analysis to check for an alternative way to introduce or embed an approval criterion^16^.

**Figure 2:**
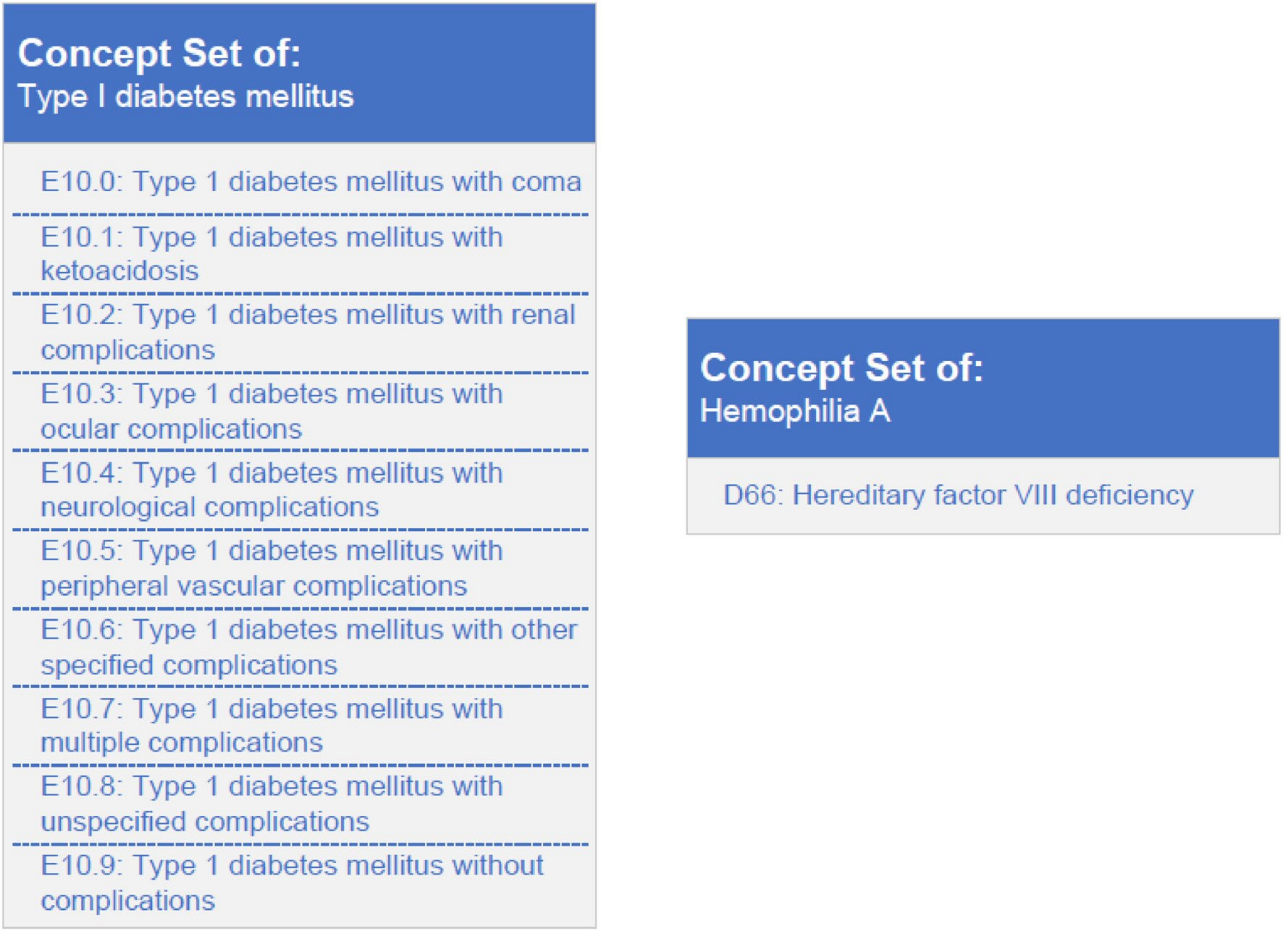
Comparison of concept sets between type I diabetes mellitus (left) and hemophilia A (right).

In the next step, the eligibility criteria were implemented in ATLAS software. We used ATLAS Version 2.9.1 in combination with OMOP CDM 5.3.1. For this purpose, the corresponding vocabulary for the chosen terminology systems was utilized in the most recent version: ICD-10-GM, OPS, LOINC, ATC and SNOMED CT from the official Vocabulary Set of OHDSI^26^. In ATLAS, all fitting codes describing a single eligibility criterion are summarized in one concept set, which bundles one or more concepts. A selection criterion corresponds to a concept set, which consists of one or several codes covered by single concepts. This is necessary because a single criterion can be composed of one to hundreds of codes^27^. For example, type I diabetes mellitus can have different characteristics, which can be marked with different ICD codes. Codes for these disease individualities create one concept set. In comparison, hemophilia A could be created as a concept set using a single code, as there is only one ICD-GM-10 code for this specific disease (Fig. 2). If there was uncertainty regarding an eligibility criterion or related codes, medical personnel were consulted. Afterward, a separate concept set had to be created for each eligibility criterion, although no concept sets were created for demographic data such as age or sex. The aforementioned criteria were entered directly during a later process within a cohort by means of the corresponding functions available in ATLAS.

Once all the concept sets required for a study were established, cohorts that included all the inclusion and exclusion criteria for a study had to be defined. To outline a cohort, it was first necessary to define an initial event, which was a main criterion and was used for the preselection of subjects. All individuals who met the requirements of the initial event comprised the main cohort of patients. To set further constraints, it was possible to set other eligibility criteria to obtain more specified results. We selected the disease under study of the trial as an initial event for this analysis. This information can be found on ClinicalTrials.gov under “Conditions”. For example, if a study has the eligibility criteria of type I diabetes mellitus, age under 18 years and elevated body mass index (BMI), we would define diabetes mellitus as the initial event. All patients with type I diabetes compose the main cohort. The criteria “age” and “BMI” would be added as further eligibility criteria.

### Categorization of eligibility criteria and studies

Of all 331 unique eligibility criteria, 108 criteria were excluded because of missing relevance. These criteria were those that have no relevance at this stage of recruitment and do not exist prior to a face-to-face interview with a study physician, such as the availability of a consent form. Only after identification of a patient who fulfils certain characteristics (such as the presence of a disease) using the electronic tool, a study physician interviews the potential subject regarding, for example, medical history, study procedure, language skills, and informed consent. For this reason, these criteria cannot be analyzed in the process of electronic patient screening.

To categorize the eligibility criteria, we analyzed the remaining 223 suitability criteria and classified them in terms of their implementation in ATLAS into the following categories: implementable, partially implementable, and not implementable (Table 1). If a criterion were only partially or not implementable, we would also add short notes expressing our decision.

**Table 1:** Eligibility criteria categories.

Criteria category	Explanation
Implementable	Criterion can be implemented in ATLAS using ICD, OPS, ATC or LOINC
Partially implementable	Criterion can be implemented using only SNOMED CT or only a part of the criterion is mapped (for example, a disease can be implemented, but a timely constraint is not possible)
Not implementable	Criterion cannot be implemented with the help of ICD, OPS, ATC, LOINC or SNOMED CT or the functions available in ATLAS

After weighting the studies with respect to their feasibility, four categories were established: fully portrayable, portrayable, partially portrayable, and not portrayable (see Table 2).

**Table 2:** Explanation of categories in the overall evaluation of the feasibility of measures taken in ATLAS.

Trial category	Explanation
Fully portrayable	All criteria of a study can be implemented in ATLAS
Portrayable	The initial event is classified as implementable and can be mapped completely in ATLAS. Further inclusion criteria can be classified as partially implementable or not implementable according to Table 1
Partially portrayable	The initial event is classified as partially implementable in ATLAS according to Table 1
Not portrayable	The initial event cannot be implemented in ATLAS

### Classification of partially/not implementable criteria

Finally, we performed a secondary analysis to discover the main difficulties in the introduction or implementation of eligibility criteria in the software ATLAS. Therefore, all reasons and arguments were considered to justify the categorization and the implementation issues of the eligibility criteria.

### Quality assurance

This study was divided into three main steps, which were conducted by two authors with a background in medical informatics. This study began with a training phase, during which both individuals independently rated and categorized the same study in ATLAS. Afterward, they discussed their results until a consensus was reached. This step was repeated until both authors independently categorized the study without collusion. In the next step, one individual performed all mapping and categorizing for all trials. Any criteria that were partially or ambiguously depicted were discussed with the other individual. Furthermore, the second author randomly checked selected trials.

### Ethical considerations

No ethical approval was needed.

## Results

The described categorization resulted in an implementation of 30 cohorts with one initial event per cohort and an additional 223 eligibility criteria. To implement all eligibility criteria and initial events, we built 129 different concept sets, including 11.20 concepts on average. Each cohort included an average of 5.33 concept sets with 59.73 concepts in total. The whole table of eligibility criteria and categorization can be found in Appendix 1.

### Categorization of eligibility criteria and studies

#### Evaluation of eligibility criteria

Of all 223 criteria, 35.19% (n = 82) were focused on a patient’s health status, 21.46% (n = 50) pertained to treatment or health care, 16.31% (n = 38) to diagnostic or laboratory tests, 10.73% (n = 25) to demographics, 0.43% (n = 1) to ethical considerations, and 3% (n = 7) addressed lifestyle choices. Additionally, 8.97% (n = 20) addressed more than one of the semantic categories and were therefore categorized as “Various”.

According to our observations, 51.12% (n = 114) of all eligibility criteria identified in the studies could be mapped in ATLAS and so were classified as implementable (see Table 1). This set corresponded with the eligibility criteria referring to clearly defined diseases, procedures, or drugs with the corresponding ICD, OPS, ATC or LOINC codes. Additionally, 21.11% (n = 47) of the criteria were defined as partially implementable, and 27.80% (n = 62) could not be implemented in ATLAS at all.

#### Evaluation of studies

We grouped the selected 30 studies after the ICD-10 chapters of their primary addressed health issue. This information was taken from their official trial description on ClinicalTrials.gov. The distribution of this grouping can be found in Table 3.

**Table 3:** Distribution of the primary addressed health issues of the selected trials.

Certain infectious and parasitic diseases (A00–B99)	1
Malignant neoplasms (C00–C97)	4
Endocrine, nutritional and metabolic diseases (E00–E90)	10
Respiratory system diseases (J00–J99)	2
Diseases of the blood and blood-forming organs and certain disorders involving the immune system (D50–D90)	3
Diseases of the circulatory system (I00–I99)	5
Diseases of the musculoskeletal system and connective tissue (M00–M99)	1
Nervous system diseases (G00–G99)	1
Diseases of the urogenital system (N00–N99)	2
Factors influencing health status and contact with health services (Z00–Z99)	1

Based on our evaluation, in 10% of the studies (n = 3), all criteria could be fully mapped, so these studies were classified as fully portrayable (see Table 2) The latter set included a few simple and clearly defined criteria, such as a well-defined disease, forming a group of subjects which could be filtered by one or two additional criteria, like age or sex. In these cases, the disease was introduced into ATLAS using the appropriate codes and as an initial event.

Additionally, 73.33% of the studies (n = 22) were classified as portrayable, where at least one of the eligibility criteria was only partially or not implementable according to the rules in Table 2. However, the initial event could be fully implemented as a concept set using ICD, OPS, LOINC or ATC. In 16.67% (n = 5) of studies, it was not possible to fully implement the initial event, and these were assigned to the partially portrayable category. Finally, none of the studies were classified as not portrayable.

### Classification of partially/not implementable criteria

#### Partially implementable criteria

After analyzing the justifications corresponding to the criteria that could not be introduced into ATLAS, we categorized them into four groups described in Table 4.

**Table 4:** Categories of partially implementable criteria.

Criteria category	Explanation
Missing information	Description is not precise
Highly specialized criterion	Highly specialized diagnosis, procedure or laboratory result
Technical reasons	Partial aspect is not feasible due to technical reasons
Terminology system	Only implementable with SNOMED CT

According to the results, 36.17% (n = 17) of the partially implementable criteria belonged to the associated class due to technical reasons, with different causes. For example, in some studies, age was indicated in years, but in others, such as pediatric studies dealing with infants, age was indicated in months. Some criteria also needed a cause-and-effect relationship. In such criteria, information regarding whether the patient is involved in cross-studies is required, which cannot be extracted from an OMOP data model.

Among the remaining criteria, 27.66% (n = 13) could not be fully implemented due to missing information. Those criteria mainly refer to “norm” or “stable” values even if there are no defined norm quantities valid in all countries or locations but different standards from site to site. The latter set includes criteria that refer to the groups of medicaments, diagnosis, procedures, or laboratory results, which are not globally defined.

**Figure 3:**
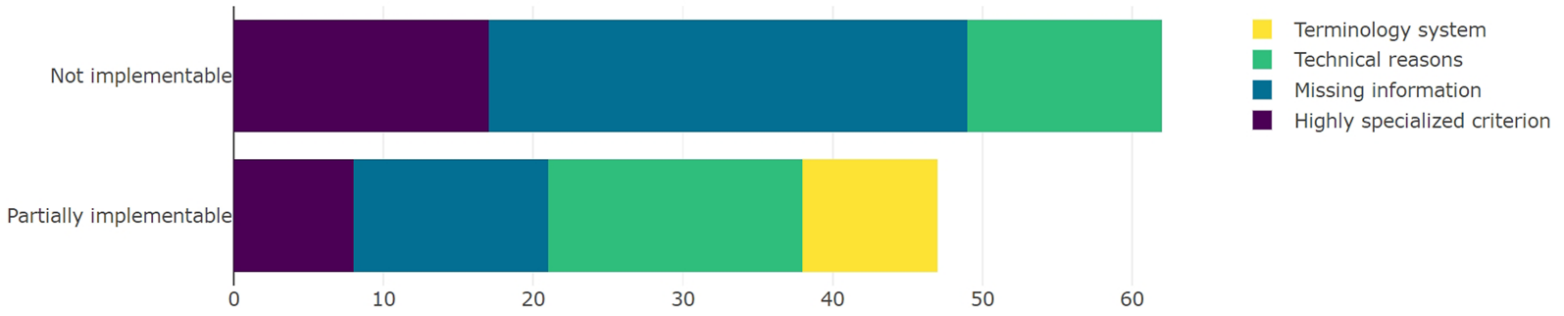
Number of eligibility criteria and implementability with respect to the reasons for categorization.

A total of 17.02% (n = 8) of criteria were too specific, and no terminology system was found to have a specific corresponding code, except for the codes that also included a group of diseases/procedures/laboratory results/medications. Finally, 19.15% (n = 9) of eligible criteria were classified with SNOMED CT. All these results are shown in Fig. 3.

#### Nonimplementable criteria

To categorize the criteria that were not implementable, we selected the same categories, without the category corresponding to the wrong terminology system. As shown in Fig. 3, in 51.61% (n = 32) of the nonimplementable criteria, missing information was the problem. A total of 27.42% (n = 17) of the criteria were too specific, and 20.97% (n = 13) were not implementable due to technical reasons.

### Semantic criteria categories

**Figure 4:**
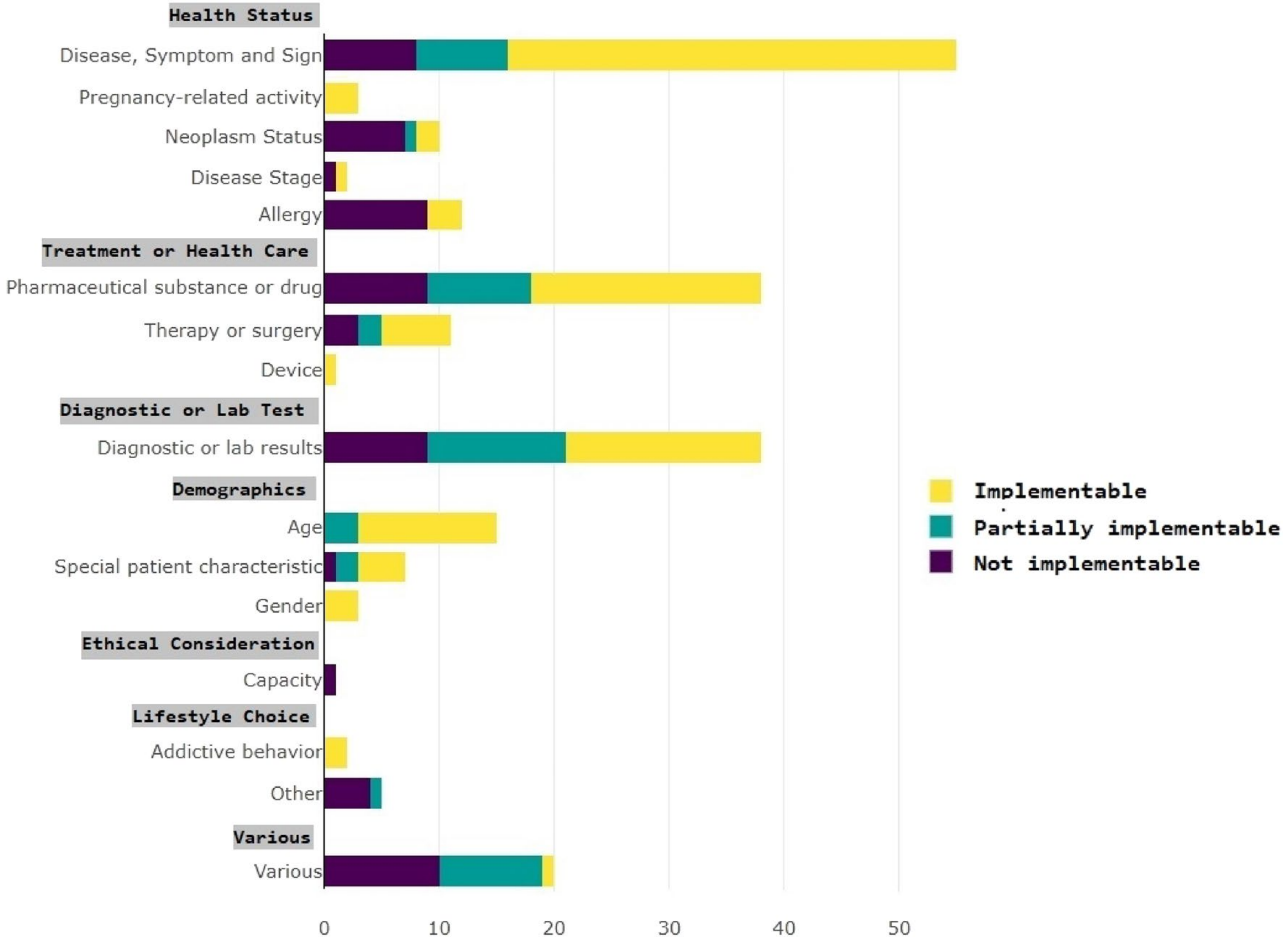
Eligibility criteria categorized with the semantic categories of Luo et al.^22^ with their degree of implementability in ATLAS. The underlying data can be found in Appendix 2.

Figure 4 shows the portraiture of ATLAS in relation to the semantic criteria categories. The different semantic categories of the inclusion criteria can be portrayed to varying degrees in ATLAS. For instance, criteria under the category "Disease, Symptom, and Sign" can be portrayed up to 60% in ATLAS, while other categories are either not portrayable or to a lesser degree.

### Technical limitations of ATLAS

ATLAS software allows for the mapping of medical criteria using various options, ensuring flexibility. Nevertheless, the software's user interface is complex, making intuitive use of it challenging, even for experienced personnel. Before commencing research, an extensive familiarization phase is needed. Additionally, individual customizations are necessary to input intricate or nested study criteria. Scores are frequently not available as a standalone code. Analyzed scores generally comprise a number of subitems, such as diseases or laboratory values, with each being assigned a value based on the severity of its impact. As a result, each aspect of a score is initially treated as a distinct criterion that must be included in the system as separate concept sets, a significant undertaking in complex-scale studies.

ATLAS has the possibility to input basic demographic data in an easy way and without assigning a concept, but these data are limited to age and sex. Other basic information, such as height and weight, must be input similar to other observations. For this purpose, the appropriate LOINC code has to be identified, created as a concept set, and time constraints might have to be set when only the latest data on height and weight are searched. At this point, this information can be integrated into the cohort.

Additionally, temporal information, such as the starting point of an event or a treatment, can only be introduced as an absolute date or relative to the time of the initial event, not as relative time information such as “six months ago”. Therefore, the selection criteria for a cohort regarding the period must be updated on a daily basis.

## Discussion

### The utilized terminology systems

Since ATLAS software is based on the involvement of terminology systems, this analysis highlights the importance of the choice of terminology systems and thus the availability of codes contained in the terminology system. The ICD-10-GM, ATC and LOINC are international classification systems widely used in German clinics in hospital information systems; therefore, they are easy to access and use if hospital data are queried. Additionally, OPS is a terminology system used only in Germany for billing code procedures^28^.

The medical terminology within SNOMED CT provides considerably more detailed diagnostics than ICD-10. Not only does SNOMED CT include diagnoses, but it also incorporates medical terms that are interrelated. In contrast, the ICD-10 is based on a hierarchically organized structure of diagnoses and is employed in over 140 countries with varying adaptations^29^. In Germany, the use of the ICD-10-GM is mandatory for billing in hospitals and primary care settings. For that reason, it is more widely utilized in patient care systems, providing ease of access. Additionally, medical personnel are accustomed to working with ICD-10-GM codes and are familiar with their strengths and weaknesses.

Nevertheless, it is important to examine the usefulness and intended audience before asserting that certain terminologies are better suited for a specific purpose. Since the ICD-10 was developed for epidemiological and billing purposes, it takes a different approach than the SNOMED CT, which is suited for the integration of eHealth applications. Utilizing a combination of coding methods, such as combining ICD and SNOMED CT, can also prove advantageous^30^.

In addition to the 51% of the terms that could be mapped by the ICD-10-GM, ATC, LOINC and OPS*, further criteria could be mapped by using SNOMED CT*^8^. Nine criteria were not possible to map by the chosen terminology systems but with the system SNOMED CT. Thus, including all SNOMED CT codes in criteria mapping can increase the coverage rate to 55%^30^.

However, there were many eligibility criteria that could not be mapped by means of chosen classification systems (neither by the ICD-10-GM, OPS, ATC, or LOINC nor by SNOMED CT). * In certain cases, a specific manifestation of a disease, procedure, or laboratory value was searched for, but only the basic information without the specifics could be entered into the system, which was represented by eight partially implementable criteria. Some criteria could not be implemented at all because the level of specification was too high (17 of the nonportrayable criteria). This is because clinical trials are often designed to gain knowledge about novel or underresearched diseases. The codes for these conditions do not yet exist in the evaluated terminology systems, which makes it difficult or impossible to introduce some selection criteria into ATLAS. To enhance this metric, incorporating additional terminology systems such as OrphaCodes, which cater to rare diseases, may be beneficial^31^.

We discovered three criteria that could only be mapped utilizing OrphaCodes, although the OMOP system had not yet integrated OrphaCodes’ terminology system at the time of analysis^32^.

Another example of this is clinical research in oncology. The study by Verwej et al. showed that oncology is characterized by constant progress due to the continuous discovery of novel and specific gene mutations in cancer screening and care^33^ This aspect was also present in this project because specific gene mutations were identified among the partially or nonimplementable eligibility criteria, and these mutations cannot be represented in ATLAS due to the current state of research.

According to our observations, we can conclude that formulated eligibility criteria have a great impact on the use of correct terms, but on the other hand, for specialized criteria, no fitting codes are available. Additionally, it has been shown that the choice and correct use of medical classification systems, such as the definition of inclusion and exclusion criteria, is an important basis for working with ATLAS. Therefore, there is a need for the correct use of terminology systems and standardization, as well as continual adaptations to medical progress, which researchers, physicians, and others involved in health care need to strive for.

In this study, the effectiveness and suitability of the individual classification systems were not analyzed. Therefore, we could not draw conclusions about the usability of some terminologies in ATLAS.

*These statements refer to the versions described in Section “Observational health data sciences and informatics (OHDSI)”.

### Process of cohort definition

The tool criteria2query (first version) can assist in generating cohort definitions based on standard concepts, but its ability to actively select specific vocabularies for cohort definition generation is limited. As the definition of an ATLAS cohort heavily relies on engaged terminology systems, these tools may not be suitable for use in the presence of other supported vocabularies^34–37^.

Criteria2query provides a considerable advantage, as it automatically assigns concepts or concept sets to criteria and maps the logic of links between individual concepts. This feature saves time in defining cohorts^34^. While the tool supports nonstandard vocabularies, using it becomes more complicated when assigning special concepts through the tool. It may even require external tools to find the appropriate codes. Hence, we abstained from utilizing criteria2query for our analysis.

### Categorization of criteria

Our results show a relatively poor coverage rate of eligibility criteria in ATLAS. Nevertheless, ATLAS is widely used in the international community to perform medical research. The number of research studies conducted with the help of OMOP CDM and ATLAS has also increased in the last five years^38^.

International research projects utilizing the OMOP CDM rely on ATLAS to establish patient cohorts and assess them based on various patient-related variables. This is performed to acquire new medical knowledge. The patient cohort's eligibility criteria have been adapted and precisely formulated for use on OMOP CDM and the current data fields. Eligibility criteria for clinical trials are not intended for technical use but rather serve as guidelines for researchers with extensive knowledge of the medical field relevant to the study. Thus, they tend to be imprecise, as our results demonstrated. Additionally, eligibility criteria are not computer-readable and are sometimes complex^39^. Another factor contributing to the low coverage rate is the fact that some eligibility criteria are too specialized to be depicted by ATLAS or described by any of the provided terminology systems. The primary reason is that eligibility criteria are defined for use by study physicians or personnel and often afford room for individual professional judgment. However, these criteria are not universally defined, and each person may have a slightly different interpretation of, for example, "stable laboratory values." Nonetheless, an accurate and unambiguous description of the criteria is crucial for a deep understanding of this process. Without a precise description of criteria, it is not possible to implement them accurately.

### Limitations of ATLAS

Training is needed to fully understand the ATLAS software. Additionally, there may be a need for making individual adaptations in some situations to introduce complex or nested datasets, such as scores. These issues highlight usability problems with ATLAS, which have been confirmed in other studies as well. In 2021, Schüttler et al. performed a qualitative exploratory study followed by a web-based usability test to evaluate three research tools. They found that ATLAS provides significantly more features than Integrating Biology and the Bedside (i2b2) developed by the National Institutes of Health or Sample Locator by the German Biobank Alliance, along with a well-designed interface. However, they found numerous usability issues arising and can be attributed to the various functions and diverse approaches for introducing selection criteria^40^.

ATLAS was primarily developed for researchers and experts who frequently formulate intricate cohort inquiries. Consequently, it is crucial to offer them a range of selection and input options, even if it results in increased operational expenses^40^.

This can present difficulties, particularly when employed in a CTRSS, as study personnel often lack the necessary time and technical expertise. Since becoming familiar with the software may be a time commitment for all study personnel, it is prudent to designate only a few personnel as ATLAS specialists for the team. For example, the European Health Data and Evidence Network (EHDEN) offers a variety of training sessions that may prove useful for this endeavor^41^. It may be helpful for study personnel to assist with implementing eligibility criteria in ATLAS by utilizing criteria2query.

It is worth mentioning that an inaccurate implementation of eligibility criteria in ATLAS can lead to less precise subject recommendations. If the entry of studies into ATLAS is intended to lead to suitable subject recruitment, the involvement of specialized study physicians or study personnel in the process of cohort definition will be helpful.

### Digital subject recruitment

OMOP CDM and ATLAS, as products of OHDSI, have been developed to support multi-institutional research projects. In this study, we intended to test the possibility of using OMOP CDM and ATLAS not only to perform analyses of the existing datasets but also to implement the eligibility criteria of clinical trials. This makes it suitable as a digital tool for recruiting candidates for clinical studies. The implementation of trial eligibility criteria in ATLAS cohorts is more complex than a 1:1 translation: approximately half of the eligibility criteria could not be fully implemented in ATLAS.

Forty-five of the analyzed criteria could only be partially mapped or not mapped at all in ATLAS due to missing descriptions of the criterion. The lack of such an understanding results in criteria not being introduced into the system or being incorrectly implemented, and subjects are often incorrectly excluded from studies. The precise formulation of subject characteristics is initially needed for clinical research, so well-chosen criteria are crucial for the feasibility and internal validity of studies^42^. In terms of ATLAS, carefully chosen and precisely formulated eligibility criteria form the basis for being able to use the software correctly and to exploit its potential functionalities. To obtain better results in the process of formalizing eligibility criteria for an automated search, it is helpful to collaborate tightly with the study personnel so that imprecise criteria can be refined by professionals. In addition, the use of correct and standard medical language is important so that the meaning of the criteria can be better understood. If everyone follows and understands given standards, such as vocabulary or terminologies, this will positively impact the use of systems for patient recruitment^43^. Subsequently, all these criteria and therefore more trials can be implemented in ATLAS.

### Outlook

As stated previously, numerous inclusion and exclusion criteria cannot be implemented in ATLAS due to insufficient information in the descriptions. Therefore, a secondary analysis should be conducted, which includes the cohort definitions alongside medical experts from the relevant clinical department. We aim to raise the rate of implementable eligibility criteria by 20%.

We aim to assess the applicability of all eligibility criteria specified in the official trial descriptions for searching real-world data. We assume that exclusion rates for certain criteria may substantially differ, and thus, we endeavor to identify the screening criteria that are most important and those that are negligible.

## Conclusion

Although clinical studies are vital for medical research, the lack of a sufficient number of participants often leads to their failure. It is a major challenge to identify eligible candidates for clinical trials. OMOP CDM, when combined with ATLAS, can be utilized to search for subjects in principle. However, we found that mapping inclusion criteria in ATLAS works seamlessly for only approximately half of the criteria. Reasons for this are often that the specified criteria are not clearly defined and for this reason cannot be fully implemented. Unclear and undefined criteria can result in the failure to implement approximately 20% of all inclusion criteria. Effective collaboration with study personnel to map out these criteria can lead to the replacement of imprecise information with unambiguous criteria, leading to substantially higher coverage rates.

Another reason criteria may not be fully implemented is that some require querying highly specialized diagnostic characteristics, laboratory values, medications, or other patient features. SNOMED CT or OrphaCodes may enable the mapping of additional criteria in certain cases. Employing other terminology systems may also prove beneficial depending on the local setting.

Finally, some of the criteria (13%, n = 30) could not be fully implemented due to the circumstances of ATLAS. Here, whether the criteria can be entered in a simpler form that is possible to implement must be evaluated in cooperation with the study staff. However, it is sometimes possible to use similar criteria that are valid for preselection. In addition, the study personnel can prioritize the relevance of different criteria, as some of the criteria are more important for a selection of candidates than others.

It is unclear at this time whether there is a prioritization of inclusion criteria that must be considered when implementing study criteria in ATLAS. It is possible that the nonimplementable criteria may also be of less relevance for the search for subjects. On the other hand, it is also conceivable that precisely these criteria that could not be mapped are particularly important. Further work is planned to examine this connection and to be able to make a more precise statement about how well ATLAS works in a real-world setting for study recruitment.

### Supplementary Information

Supplementary Table 1.

Supplementary Table 2.

## Data Availability

All data generated or analyzed during this study are included in this published article and its supplementary information files.

## Abbreviations

ATC: Anatomical therapeutic chemical classification
BMI: Body mass index
CDISC: Clinical data interchange standard consortium
CDM: Common data model
CTRSS: Clinical trial recruitment support system
ICD: International statistical classification of diseases and related health problems
ICPM: International classification of procedures in medicine
i2b2: Informatics for integrating biology and the beside
LOINC: Logical observation identifiers names and codes
OHDSI: Observational health data sciences and informatics
OMOP: Observational medical outcomes partnership
OPS: Operation and procedure code
PRS: Patient recruitment system
SNOMED CT: Systematized nomenclature of medicine clinical terms
